# Supplementary material for: Tear Break-Up Time and Seasonal Variation in Intraocular Pressure in a Japanese Population
Source: Diagnostics (Basel). 2020 Feb 24;10(2):124. doi: 10.3390/diagnostics10020124 (PMC7167993; doi:10.3390/diagnostics10020124)
Supplement: Supplementary file 1 [file diagnostics-10-00124-s001.pdf]

Table S1. Glaucoma medications in each group.

		Glaucoma without short BUT	Glaucoma with short BUT
# of medication	1	28	27
	2	7	5
	3	1	4
	Mean*	1.4 ± 0.7	1.2 ± 0.5
Frequency of instillation	1	27	27
	2	4	1
	3	3	4
	4	1	1
	5	1	3
	Mean**	1.4 ± 1.0	1.7 ± 1.3
Medications PG	Latanoprost	21	20
	Tafluprost	8	11
	Travoprost	3	3
	Bimatoprost	2	2
Fixed combination	PG/timolol	2	6
	CAI/timolol	5	6
Beta blocker	Timolol	10	15
	Carteolol	0	1
CAI	Dorzolamide	5	5
	Brinzomide	0	3
Other[A]		0	4

[A] = Brimonidine tartrate for three and ripasudil hydrochloride hydrate for one. Abbreviations; BUT, tear break-up time; PG, prostaglandin analogue; CAI, carbonic anhydrase inhibitor. *P = 0.342, **P = 0.416 (Kruskal-Wallis).

All of prescribed glaucoma medications contained benzalkonium chloride or other preservative; SofZia in travoprost eyedrop and Polyquad in fixed combination with travoprost and timolol maleate. Active compounds of glaucoma medication prescribed in the present study are as follows: 0.005% latanoprost, 0.0015% tafluprost, 0.004% travoprost, 0.003% bimatoprost, fixed combination of 0.005% latanoprost and 0.5% timolol maleate, fixed combination of 0.0015% tafluprost and 0.5% timolol maleate, fixed combination with 0.004% travoprost and 0.5% timolol maleate, fixed combination with 0.5% timolol maleate and 1% dorzolamide hydrochloride, 1% brinzolamide, 1% dorzoramide hydrochloride, 0.1% brimonidine tartrate, 2% carteolol hydrochloride, 0.4% ripasudil hydrochloride hydrate, and 0.5% timolol maleate.

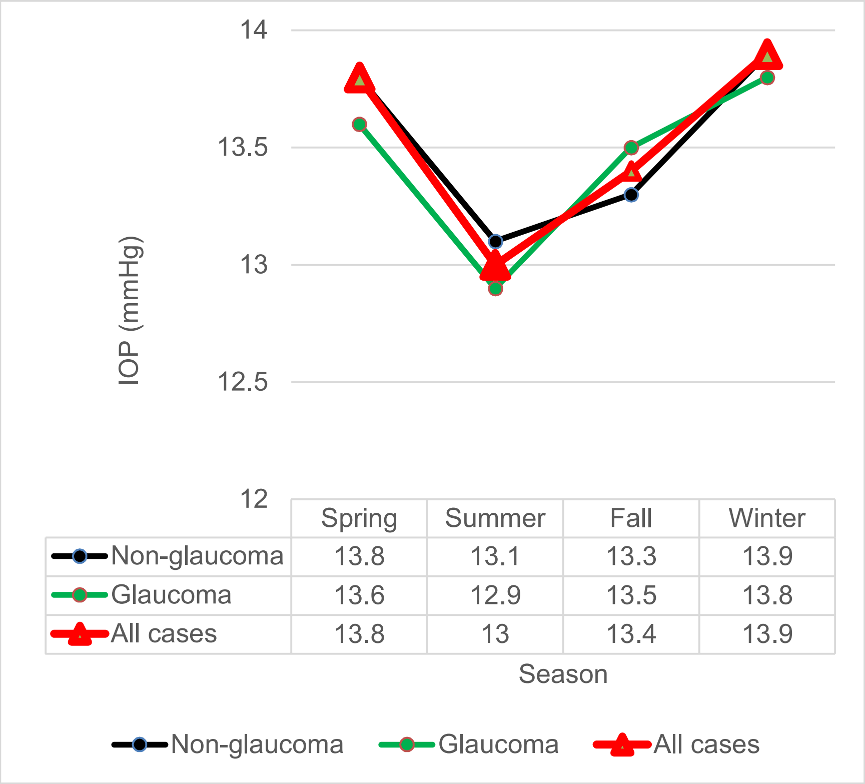

Figure S1. Intra-ocular pressure (IOP) of glaucoma and non-glaucoma groups. Seasonal IOP fluctuation before grouping clearly indicates that seasonal IOP may be IOP behavior in short BUT (tear break-up time) cases as shown in Fig S1.
